# Structures for the care of people with dementia: a European comparison

**DOI:** 10.1186/s12913-022-08715-7

**Published:** 2022-11-18

**Authors:** Tim Schmachtenberg, Jessica Monsees, Jochen René Thyrian

**Affiliations:** 1 Deutsches Zentrum für Neurodegenerative Erkrankungen e.V. (DZNE), Site Rostock/Greifswald, Ellernholzstraße 1-2, 17489 Greifswald, Germany; 2 Present Address: Present Address: Universitätsmedizin Göttingen, Institut für Allgemeinmedizin, Humboldtallee 38, 37075 Göttingen, Germany; 3 Universitätsmedizin Greifswald, Institut für Community Medicine, Ellernholzstraße 1-2, 17489 Greifswald, Germany

**Keywords:** Dementia, Care, Structures, Services, Models of good practise, Gaps, Europe, Expert interviews

## Abstract

**Background:**

Dementia is a disease that impacts people with dementia, their families, and the healthcare system. In 2018, the number of people with dementia in the EU, the European Free Trade Association (EFTA), and the UK was estimated to be 9.1 million. National dementia strategies and publications by organisations such as Alzheimer Europe outline how dementia-specific care should be designed. This study aims to provide insights into existing formal care structures, models of good practise, and gaps in dementia-specific care for people with dementia in 17 European countries.

**Methods:**

The research is based on guided interviews with country-specific care experts. A mixed-methods approach with a combination of open and closed questions was used. All interviews were recorded and transcribed verbatim based on the transcription rules of Kuckarts (2010). For data evaluation, the qualitative content analysis model of Mayring (2014) was used.

**Results:**

In all 17 countries, efforts for dementia-friendly care and models of good care practise exist. However, there are large differences between European countries regarding the spread of dementia-specific services. In nine countries (Bulgaria, Finland, Italy, Liechtenstein, Luxembourg, the Netherlands, Norway, Sweden, the UK), there are already nationwide structures, while in five countries (Belgium, Greece, Ireland, Portugal, Romania), services are only available in certain regions. In three countries (Austria, Denmark, Germany) dementia-specific outpatient services are widespread nationwide, whereas inpatient services are not. Simultaneously, in all countries, areas with major care gaps exist. Several European states have an urgent need for action concerning the expansion of the provision of dementia-specific services, the reduction of regional differences regarding the provision of care, the elimination of barriers to access to care, the dementia-friendliness of services, and the participation of people with dementia and their relatives in care and research.

**Conclusions:**

To reduce the existing structural inequalities in care between and within European countries, and to establish quality-related minimum standards in the care of people with dementia, transnational concepts are needed. The EU, in cooperation with care planners, research institutions, care providers, and patient organisations, should develop European care guidelines or dementia plans that contain concrete measures, schedules, and budgets.

**Supplementary Information:**

The online version contains supplementary material available at 10.1186/s12913-022-08715-7.

## Background

Dementia is a disease with a comprehensive impact on people with dementia, (caring) relatives, and the healthcare system. People with dementia may experience limitations not only in cognitive abilities and behaviour [1] but also, for example, depression and anxiety [2]. Dementia is often accompanied by high rates of unmet needs. One study shows that people with dementia can have eight or even more unmet needs on average. The needs areas are pharmacological treatment and care, safety at home, social counselling and legal support as well as dehydration and poor nutrition. The needs are mostly associated with indicators of need for care but also socioeconomic and demographic factors. Furthermore, they are influenced by caregiver’s demographic and socioeconomic status and their need for care [3, 4]. The severity of dementia, behavioural problems, or psychological symptoms of people with dementia can be a cause of depression and a great burden on caregivers [5]. The literature suggests that there are unmet needs among those caring for people with dementia. A study conducted in Germany determined a number of up to six or in some cases even more unmet care needs for family caregivers. These unmet needs arise in areas such as physical and mental health or quality of life [6, 7].

Alzheimer Europe estimates in its ‘Dementia in Europe Yearbook 2019’ the number of people with dementia at approximately 9.1 million in 2018 for the EU^1^, the EFTA^2^, and the UK^3^. By 2050, this number is estimated to rise to approximately 16.8 million [8]. In 2015, this high number of people affected outcomes in annual costs for the treatment and care of Alzheimer’s disease in the EU-28 of €119.6 billion (for mild stages of the disease), €66.8 billion (moderate), and €45.6 billion (severe). These costs are estimated to increase to €330.4 billion (mild), €180.8 billion (moderate), and €121.3 billion (severe) by 2050 [9].

On a policy level, European countries are increasingly publishing national dementia plans (NDPs) in which they outline various areas that are relevant for the dementia-specific care of people with dementia and their caregivers [10]. There are also reports from organisations such as Alzheimer Europe, Alzheimer’s Disease International, and Alzheimer societies on how best to provide dementia-specific care. However, in practise, the care situation of people with dementia and their caregivers may be different from what is required and desired in NDPs and reports from these organisations. Furthermore, not all European countries have a NDP and there is a wide variation in the actuality of such documents. Some countries (for example Norway) regularly update their dementia action plans, while there are other countries whose NDPs are more than 5 years old.

Although the organisation of healthcare in the EU is the responsibility of individual countries, the European Commission’s objectives include protecting and improving the health of Europe’s inhabitants, making health systems more resilient, and supporting healthcare systems to modernise and digitalise. To implement these goals and make countries’ healthcare systems accessible, resilient, and effective, the European Commission’s Directorate-General for Health and Food Safety provides aid in the form of funding, coordinating exchanges between EU countries and health experts, or through health promotion activities. On this basis, it makes sense to develop an EU strategy to support people with dementia and their families, which individual states can use as a guideline [11].

The aim of this study is therefore to provide national and international care planners with insights in available formal dementia-specific support and care structures in different European countries, existing models of good care practise in Europe, and areas of dementia-specific care with gaps that create a particular need for action. By systematically presenting these insights in existing structures, models of good practise, and gaps in dementia care at the national and European levels, care planners will be shown fields of action as well as potential solutions and networking opportunities.

This study explicitly asks for the existence of dementia-specific care structures because the authors argue that, due to the major impact of a dementia condition on the living situation of people with dementia and their family members and the resulting specific needs of these people, there is a necessity for specific services tailored to these needs. Simultaneously, it is important to make the general services of health and care systems dementia-sensitive, as people with dementia still have many needs in common with people without dementia and it is important and desirable for their social and societal inclusion as well as for the quality and diversity of societies that they participate in general services. This, in turn, can lead to increased sensitivity to dementia and the needs of people with similar conditions on the side of care systems and societies.

## Methods

The study presented in this paper is a mixed-methods approach with qualitative and quantitative elements. To collect the data, guided interviews with experts from different European countries were conducted. This study aimed to interview at least one national expert on dementia care and at least one expert on dementia and migration for as many EU, EFTA, and UK countries as possible about existing dementia-specific care structures. The guide included both open questions and closed questions with standardised answer categories. The interviews, which were conducted mainly via the Zoom video conferencing platform, were recorded, transcribed, and finally evaluated. In evaluating the data, content analyses were conducted. The model of qualitative content analysis by Mayring (2014) was used for orientation.

This study was conducted within the framework of the project ‘EU Atlas: Dementia & Migration’ funded by the Robert Bosch Foundation. Experts in the fields of health and dementia care, as well as migration, were interviewed in n = 17 European countries. The participants were researchers, care planners, care providers, or representatives of dementia associations or Alzheimer societies and thus general experts in the field. They were asked questions about formal care of people with dementia and the support of their family members, some specific to the care of people with a migratory background, and some aiming at the general population. While largely comprehensive responses to migration-specific items have been published, general population data are systematically published for the first time in this article.

## Recruitment of study participants

Experts were recruited through professional events such as the Alzheimer Europe Conference 2019 or the Public Health Conference 2019. The eligible participants of these conferences were personally contacted on site. In addition, authors of dementia-specific articles identified via databases such as PubMed, editors, and authors of NDPs and care guidelines, as well as representatives of national ministries of health, professional societies, and Alzheimer societies, were contacted and invited to participate in the interviews by e-mail. Study participants were purposively selected based on professional expertise evidenced by publications. Overall, recruitment was a challenge exacerbated by the COVID-19 pandemic. Correspondence with some experts broke off temporarily or permanently during the pandemic. Furthermore, no experts could be recruited from several countries. A major reason for this is the fact that ‘Dementia & Migration’ is either not a topic of interest or it is such a new topic in these countries that there might not be people considering themselves to be experts in this area [12].

## Interview setting and study participants

The first interview took place in a face-to-face setting at the institution of the expert, while the remaining interviews were conducted via Zoom video conferences due to increased health risks and travel restrictions in the wake of the COVID-19 pandemic. The period over which the interviews were conducted was twelve months (early 2020 to early 2021). A total of 26 experts from 17 countries were interviewed: Austria, Belgium, Bulgaria, Denmark, Finland, Germany, Greece, Ireland, Italy, Liechtenstein, Luxembourg, the Netherlands, Norway, Portugal, Romania, Sweden, and the UK. Except for Sweden, whose expert provided written answers, all interviews were conducted orally. Almost all interviews were held in English. Exceptions are interviews with experts from Germany, Liechtenstein, and Luxembourg, which were carried out in German [12]. During the interviews, exclusively two researchers and the participants were present. The interviewers were two PhD students from the research institution ‘Deutsches Zentrum für Neurodegenerative Erkrankungen’ (DZNE), in most cases these were the two first authors of this article and in some cases, one of the two first authors was supported by another PhD student of the DZNE. Before conducting the study, the interviewers and authors of this article established professional contact with the interview participants, which included providing participants with background information about the researchers and the aims of the study.

## Interview guide

The questions for the interviews were derived from scientific articles, national and international reports, and an analysis of NDPs. For this purpose, a literature search was conducted. The results of this literature search were systematically screened for aspects relevant to migration. To avoid limiting the pool of possible topics and questions too much from the outset, not that many exclusion criteria were defined. A primary source for the questions was the Alzheimer Europe report ‘The development of intercultural care and support for people with dementia from minority ethnic groups’ from 2018 [13]. The questions covered the following areas: general information on dementia and migration (such as the importance and needs of this group), care (e.g. nationwide availability of dementia-specific health care services, suitability of these services for the care of people with dementia), inclusion and information of people with a migration background (like level of inclusion of people with dementia (with and without a migration background) into the healthcare system), professional care (such as culturally sensitive care as part of the professional qualification of healthcare professionals) and support of family caregivers (e.g. differences in information and healthcare services for family caregivers of people with dementia with and without a migration background). The interview guide developed was presented at an expert workshop, which was attended by some interview participants, and revised following expert feedback. The questions that are relevant to this study can be found in the chapter ‘Supplementary information’ at the end of this article.

Before the interviews took place, the experts were sent the interview guide with these and other questions on care, inclusion, and information of people with dementia with and without a migratory background, professional care, and support for family caregivers, together with a document containing definitions of key terms such as inpatient and outpatient care or inclusion of people with dementia and the research proposal of this project. This enabled the experts to prepare specifically for the interview and the individual questions and to obtain knowledge from other experts that they did not have themselves. As a token of appreciation for taking the time and making the effort to participate in the interviews as well as for their extensive preparatory work, the experts were offered an honorarium of €400 [12].

## Transcription and analysis of the data

The interviews, which had an average duration of 90 min, were recorded and then transcribed. Therefore, the transcription rules of qualitative content analysis by Kuckartz (2010) were applied, and verbatim transcription was carried out [14]. The evaluation of the interviews was based on the method of qualitative content analysis by Mayring (2014). A combination of deductive and inductive categorisation was used to structure the content [15]. First, two categories were deductively derived from the categories of the interview guide: (1) care services for people with dementia; and (2) inclusion and information of people with dementia and their family members. The text passages that directly referred to one of these two topics were assigned to the categories and extracted. Then, subcategories were inductively derived from the data. The next steps included the formation of a category system, sorting and summarising the material, and tabulating the data. Finally, the results were presented in the form of country profiles. For a comparative analysis of the European countries involved, the country-specific results were first summarised in a table. The data were coded again (inductively), organised within the framework of a category system, and quantitatively processed and sorted. Subsequently, the similarities and differences regarding the country-specific results were described.

## Definition of inpatient and outpatient care

In this study, inpatient care was defined as permanent care, treatment, and accommodation of a person needing care in a hospital or a nursing facility, such as nursing homes, hospices, and rehabilitation facilities. Outpatient care is to be understood as support for people in need of care and their family by providing medical or non-medical care in their residence.

## Results

An overview of the participants, their background. and which country they are from can be found in Table 1.

**Table 1:** Participants and background

Country	No. of participants	Background
Austria	1	• Head of a migration & health center
Belgium	2	• Researchers on aging and diversity
Bulgaria	2	• Board member of an Alzheimer association • Member of an Alzheimer association
Denmark	1	• Neuropsychologist and researcher on minority ethnic groups
Finland	1	• Specialist in multicultural memory work
Germany	1	• Professor and researcher for ‘Interprofessional action’ and ‘Diversity and intersectionality’
Greece	1	• Head of a mental health unit for migrants and refugees
Ireland	1	• Family carer of a person with dementia
Italy	1	• Neurologist and researcher on e.g. cognitive disorders in migrants and ethnic minorities
Liechtenstein	2	• Psychologist and board member of an Alzheimer association • Nursing scientist at a health care organization
Luxembourg	1	• Director of a nursing home
The Netherlands	2	• Clinical psychologist at a hospital • Geriatrician at a hospital
Norway	3	• Researcher on public health and nurse at a unit for migration and health • Member of an immigrant community • Medical doctor and professor
Portugal	4	• Psychiatrist, therapist, professor of medical psychology and behavioural medicine, researcher • Professor and coordinator of a public health research center • Health sociologist in dementia psychosocial research • Social worker from a public body of the ministry of health
Romania	1	• Member of an Alzheimer association
Sweden	1	• Researcher on clinical memory research
UK	1	• Professor at an institute for aging and memory

The results are first presented separately in tabular form (see Tables 2, 3, 4, 5, 6, 7, 8, 9, 10, 11, 12, 13, 14, 15, 16, 17 and 18**)** based on profiles for each of the 17 European countries considered before a comparison was made, and the situation at the European level is described. The following country profiles are in alphabetical order.

## Country profiles: the EU, the EFTA, and the UK

**Table 2:** Austria

Population (2021)	8.9 million [16]
**Area (2021)**	83,900 km² [17]
**Spread of dementia-specific outpatient care services for people with dementia**	Nationwide
**Expert notes regarding outpatient care**	In urban areas, the offer is greater than in rural areas. Larger cities such as Vienna and Graz have geriatric centres, memory clinics, and other facilities that focus on early detection, diagnosis, and preventive measures regarding dementia. However, there are also gerontopsychiatric centres in rural areas and dementia self-help groups at the municipal level.
**Spread of dementia-specific inpatient care services for people with dementia**	In urban areas
**Expert notes regarding inpatient care**	The urban-rural disparity is much greater than in outpatient care. The responsible district hospitals are poorly equipped in terms of dementia care, as they have no specialists or psychiatry services. A number of specialised facilities offer high-quality dementia care. However, these services are not sufficient to meet existing needs.
**Expert conclusion regarding dementia-specific care structures**	The structures are insufficiently developed, while the need for dementia-specific care is growing due to demographic change.
**Existing models of good practise**	1. The nationwide availability of multilingual information on dementia, 2. the system of outreach by home care nurses, 3. the legal possibility of professionalising the role of a family caregiver, including the associated entitlement to a salary, holidays, and paid rehabilitation

**Table 3:** Belgium

Population (2021)	11.6 million [18]
**Area (2021)**	30,500 km² [19]
**Spread of dementia-specific outpatient care services for people with dementia**	Nationwide (in every municipality)
**Spread of dementia-specific inpatient care services for people with dementia**	In several regions
**Expert notes regarding inpatient care**	There are differences in terms of region and type of service. Concerning state and private nursing homes, the offer is very large, while the availability of other types of inpatient care, such as day-care facilities, is significantly lower. This is related to the low level of state support in this area and the focus of care policy on nursing homes.
**Expert conclusion regarding dementia-specific care structures**	In Belgium, there is a lack of a minimum standard for the care of people with dementia. Whether dementia-sensitive care is offered to people with dementia depends on individual organisations. Some organisations try to provide person-centred care and offer dementia-specific services, while some organisations do not. GPs and diagnostic centres are often dementia-sensitive, but this is not the norm in general hospitals. Among care providers and professional caregivers, there are often large knowledge gaps in terms of dealing with people with dementia. Another problem is access to information, which is a major challenge for people with dementia and their relatives due to the distribution of services among different organisations and websites.
**Existing models of good practise**	Projects of individual organisations such as dementia-friendly hospitals or nursing homes

**Table 4:** Bulgaria

Population (2021)	6.9 million [20]
**Area (2021)**	110,900 km² [21]
**Spread of outpatient care services for people with dementia**	Almost nationwide
**Expert notes regarding outpatient care**	Doctors, psychiatrists, neurologists, hospitals, and medical centres can be found in every region.
**Spread of inpatient care services for people with dementia**	Almost nationwide
**Expert notes regarding inpatient care**	There are nursing homes provided by the state and municipalities, in addition to private homes and hospices. Even if these offers exist and are suitable for the care of people with dementia, they are nevertheless not available in sufficient quantity, as their accessibility remains limited for people from rural regions, for example.
**Existing models of good practise**	1. The Alzheimer café, 2. the online consultation website and the Facebook page of the ‘Foundation Compassion Alzheimer Bulgaria’, 3. the availability of written informational materials

**Table 5:** Denmark

Population (2021)	5.8 million [20]
**Area (2021)**	43,100 km² [21]
**Spread of dementia-specific outpatient care services for people with dementia**	Nationwide
**Spread of dementia-specific inpatient care services for people with dementia**	No specialised services
**Expert notes regarding inpatient care**	This is due to the focus on making hospitals and similar facilities dementia-friendly instead of creating separate services.
**Expert conclusion regarding dementia-specific care structures**	Memory and outpatient clinics specialising in dementia are available in each of the five Danish regions. The responsibility for diagnosis and assessment is with the memory clinics. After a diagnosis of dementia has been given, the person is transferred over to public care. This means a dementia coordinator (there is one in every municipality) receives information about the patient and, if desired, makes contact to determine where there is a possible need for help. This also means that people with dementia are almost completely integrated into the healthcare system.
**Existing models of good practise**	1. The available dementia-friendly hospitals, 2. the nationwide provision of information about dementia in different languages by memory clinics and the Alzheimer Society, 3. the increasingly use of PPIs ^a^ for several years (for example, the Alzheimer’s Society has set up a panel with people with dementia that serves as an advisory body to many projects on dementia.)

^a^ Patient and Public Involvement.

**Table 6:** Finland

Population (2021)	5.5 million [22]
**Area (2021)**	338,100 km² [21]
**Spread of dementia-specific outpatient care services for people with dementia**	Nationwide
**Expert notes regarding outpatient care**	Home care is the most common form of treatment for people with dementia in Finland.
**Spread of dementia-specific inpatient care services for people with dementia**	Nationwide
**Expert notes regarding inpatient care**	Hospitals with specialised care services and special care partners for university hospitals exist nationwide. However, regional hospitals and university hospitals offering specialised care for people with dementia can be far away.
**Expert conclusion regarding dementia-specific care structures**	Dementia is a topic with very high nationwide attention in Finland and the existing services are suitable for adequate care of people with dementia. However, there are regional and individual differences in the implementation of treatment. This entails, for example, care in the capital, outpatient treatment, and rehabilitation plans.
**Existing models of good practise**	1. A large amount of information and materials about dementia (Consequently, dementia-specific knowledge about dementia in the general population is quite high.), 2. the strong involvement of family members of people with dementia in the assessment of services

**Table 7:** Germany

Population (2021)	83.1 million [23]
**Area (2021)**	357,000 km² [21]
**Spread of dementia-specific outpatient care services for people with dementia**	Nationwide
**Expert notes regarding outpatient care**	Outpatient nursing services, in particular, are very widespread, while outpatient GP and geriatric, psychotherapeutic, and rehabilitative care for people with dementia in rural areas is critical.
**Spread of dementia-specific inpatient care services for people with dementia**	In several regions
**Expert notes regarding inpatient care**	Facilities such as dementia units or structures such as dementia files are not yet available in all hospitals. As general somatic units often lack geriatric knowledge, care for people with dementia in hospitals is often inadequate.
**Expert conclusion regarding dementia-specific care structures**	Services for people with dementia often lack sensitivity to the specific needs of these people. The number of dementia-friendly specialists and general practitioners is far too low. Furthermore, too many people with dementia drop out of the informational and counselling system due to a lack of specific information for people with other diversity characteristics such as early-onset dementia, a migratory background, or homosexual orientation. In addition, participatory care and research with people with dementia and their relatives rarely occur. Overall, good offers exist, but a different structuring of the care system is needed.
**Existing models of good practise**	1. The concept of dementia-friendly hospitals, 2. the high activity of Alzheimer societies at the national and regional levels, 3. the almost nationwide availability of comprehensive information for people with dementia

**Table 8:** Greece

Population (2021)	10.7 million [20]
**Area (2021)**	132,000 km² [21]
**Spread of dementia-specific outpatient care services for people with dementia**	In some regions
**Expert notes regarding outpatient care**	People who have financial resources use services from the private sector or hire a person - usually with a migratory background - to live with and care for people with dementia.
**Spread of dementia-specific inpatient care services for people with dementia**	Almost exclusively in the private sector
**Expert notes regarding inpatient care**	The private sector offers many options for residential care for people with dementia, but there are hardly any alternatives outside these offers.
**Expert conclusion regarding dementia-specific care structures**	When people receive a dementia diagnosis, they have to determine for themselves which services exist and what they can use. This process can be facilitated by the nationwide availability of information for people with dementia and family members, which is promoted through different communication channels in different campaigns. People with dementia and their families are rarely involved in the creation of information, care, and support services. Although the National Dementia Action Plan calls for the participation of this group in everything related to dementia, few people with dementia and/or family members are involved.

**Table 9:** Ireland

Population (2021)	5 million [20]
**Area (2021)**	70,300 km² [21]
**Spread of dementia-specific outpatient care services for people with dementia**	In some regions
**Expert notes regarding outpatient care**	These services are mainly provided in urban areas, whereas rural areas do not offer services in this field. However, due to the COVID-19 pandemic, some memory cafés have virtualised their services so that people from rural areas can also access them.
**Spread of dementia-specific inpatient care services for people with dementia**	No specialised services
**Expert conclusion regarding dementia-specific care structures**	Existing services are not suitable to adequately care for people with dementia, as there is a lack of dementia-specific services and existing ones are often not dementia-friendly. Further problems are the difficulty in obtaining a dementia diagnosis and the dependence of access to care services on one’s place of residence.
**Existing models of good practise**	The provision of information about dementia to people with dementia and their families by GPs, community nurses, and ‘The Alzheimer Society of Ireland’ (the Alzheimer Society offers both written informational packages and a telephone counselling service.)

**Table 10:** Italy

Population (2021)	59.3 million [20]
**Area (2021)**	301,300 km² [21]
**Spread of dementia-specific outpatient care services for people with dementia**	Nationwide
**Spread of dementia-specific inpatient care services for people with dementia**	Nationwide
**Expert notes regarding inpatient care**	In Italy, there are over 600 memory clinics.
**Expert conclusion regarding dementia-specific care structures**	Existing services are generally adequate for the care of people with dementia. Information for people with dementia and their family members is available nearly nationwide. However, there is a north-south difference in the sense that there may still be a lack of services and information in the south, and that the organisation of services is not yet optimally set up.
**Recommended model of good practise**	A working group should be established that includes associations for patients and family caregivers.

**Table 11:** Liechtenstein

Population (2019)	39,000 [24]
**Area (2019)**	160 km² [24]
**Spread of dementia-specific outpatient care services for people with dementia**	Nationwide
**Expert notes regarding outpatient care**	The ‘Familienhilfe Liechtenstein e.V.’ is responsible for outpatient care. For 10 of the 11 municipalities, the ‘Familienhilfe’ provides nursing and care services as well as meal services.
**Spread of dementia-specific inpatient care services for people with dementia**	Nationwide
**Expert notes regarding inpatient care**	The ‘Liechtensteinische Alters und Krankenhilfe’ (LAK) is responsible for inpatient care. The ‘LAK’ operates 6 nursing homes at 5 locations and provides holiday, day, night, rehabilitative, and inpatient long-term care for 10 municipalities. In one municipality, there is separate follow-up care and support infrastructure, as well as a day-care service for people with dementia by the ‘Lebenshilfe Balzers e.V.’.
**Expert conclusion regarding dementia-specific care structures**	The existing care services are suitable for adequate care of people with dementia and information on dementia and dementia-specific care is available nationwide. While outpatient and inpatient services are well accepted, day-structured and night-time services for people with dementia to relieve the burden of family caregivers are not yet fully utilised. Furthermore, there is a need to improve the participation of people with dementia and their relatives in the development of services.
**Existing models of good practise**	1. The awareness-raising (e.g. through various media appearances, the biannual mailing of information brochures to all households, and digital services) and training efforts of ‘Dementia Liechtenstein’, 2. the care and support services of the ‘Familienhilfe’, 3. the nationwide programme for **engaging** people with cognitive impairment

**Table 12:** Luxembourg

Population (2021)	635,000 [20]
**Area (2021)**	2,600 km² [21]
**Spread of dementia-specific outpatient care services for people with dementia**	Nationwide
**Spread of dementia-specific inpatient care services for people with dementia**	Nationwide
**Expert notes regarding outpatient and inpatient care**	In Luxembourg, a new law was passed that requires at least 40% of staff in inpatient and outpatient settings to receive 40 h of psychogeriatric training. This measure enables the majority of staff to work adequately with people with dementia.
**Expert conclusion regarding dementia-specific care structures**	The existing care services are adequate to provide appropriate care for people with dementia and dementia-specific informational services are available nationwide. In addition to the work of the ‘Info-Zenter Demenz’ and the high presence of the ‘Association Luxembourg Alzheimer’, this is due to the efforts of the government, which emphasises in the design of training that its employees receive information about dementia that they can pass on to people with dementia and their relatives.
**Existing models of good practise**	The educational work of the ‘Info-Zenter Demenz’, which provides information about dementia in seven languages

**Table 13:** Netherlands

Population (2021)	17.5 million [20]
**Area (2021)**	41,500 km² [21]
**Spread of dementia-specific outpatient care services for people with dementia**	Nationwide
**Spread of dementia-specific inpatient care services for people with dementia**	Nationwide
**Expert notes regarding inpatient care**	Almost every hospital has a neurologist or geriatrician to contact in case of dementia symptoms. Furthermore, there are almost 100 memory clinics in the Netherlands. The larger cities also all have inpatient care facilities. In rural areas, the distances to such facilities can sometimes be slightly longer.
**Expert conclusion regarding dementia-specific care structures**	The existing care services are suitable for adequate care of people with dementia and dementia-specific informational services are available throughout the country. Since most of the information provided mainly by ‘Alzheimer Netherlands’ and ‘Pharos’ is available online, access is difficult for many older people. Consequently, there is a need for different forms of informational services. Currently, people with dementia and their relatives are rarely involved in the development of care services. One reason for this is that GPs, who play a key role in the Dutch care system, have difficulties in diagnosing dementia.
**Existing models of good practise**	The concept of the ‘dementia-friendly community’, which includes a quality label and staff training for supermarkets and communities, as well as needs assessment through biography work with people with dementia

**Table 14:** Norway

Population (2021)	5.4 million [20]
**Area (2021)**	385,200 km² [25]
**Spread of dementia-specific outpatient care services for people with dementia**	Nationwide
**Expert notes regarding outpatient care**	Home care is available to all people living in Norwegian communities. These services are almost exclusively offered by public service providers. Norway was one of the first countries to establish a dementia action plan. Since then, the government has published three NDPs. As a result of the NDPs, many municipalities have installed special dementia teams and dementia coordinators.
**Spread of dementia-specific inpatient care services for people with dementia**	Nationwide
**Expert conclusion regarding dementia-specific care structures**	The existing care services are suitable for adequate care of people with dementia and informational services for people with dementia and their family members are widespread. Through the NDPs, as well as the work of the ‘Norwegian Centre for Ageing and Health’ and the ‘Centres for Care Research’, there has been a strong focus on dementia at the national, regional, and local levels. However, the pressure on the care system is growing due to the increasing number of older people. Since the system is bureaucratic and has precise requirements on how to articulate needs, relatives often have to be resourceful to obtain the services they are entitled to. Overall, relatives need more support, for example, in the form of home-based services. Over the past decade, there has been a great focus on the care of people with dementia with action plans, the funding of research, and the development of initiatives. However, the greatest challenge is the implementation of existing knowledge in practise.
**Specifics of the healthcare system**	In contrast to countries such as Germany, Norway has a unified healthcare system and provides universal healthcare.
**Existing models of good practise**	1. the relatively high level of state investment in dementia care, 2. the broad public campaigns on dementia, 3. the increasing participation of people with dementia and their relatives in the design of care services, 4. the establishment of a dementia hotline by an organisation for people with dementia and their relatives

**Table 15:** Portugal

Population (2021)	10.3 million [20]
**Area (2021)**	92,100 km² [21]
**Spread of dementia-specific outpatient care services for people with dementia**	In some regions
**Spread of dementia-specific inpatient care services for people with dementia**	In some regions
**Expert notes regarding outpatient and inpatient care**	Reasons for the low distribution of dementia-specific services include a lack of funding and organisation.
**Expert conclusion regarding dementia-specific care structures**	The existing care services are only partly suitable to adequately care for people with dementia and there are various barriers of access to these services. Additionally, it does not seem clear who is responsible for the care of people with dementia. Currently, neurology, psychiatry, and mental health teams share this work. Informational services for people with dementia and their family members exist in some regions. These services are provided by Alzheimer cafés, the website of the ‘Association Alzheimer Portugal’, and a dementia-specific movement at the community level.
**Existing models of good practise**	A growing movement in the municipalities to reach people with dementia, raise awareness of dementia, and disseminate information.

**Table 16:** Romania

Population (2021)	19.2 million [20]
**Area (2021)**	238,400 km² [21]
**Spread of dementia-specific outpatient care services for people with dementia**	In several regions
**Expert notes regarding outpatient care**	In university centres of large cities, there is a special service for people with dementia. Otherwise, outpatient services are available where people with dementia can ask for help, but these services are not specialised for their needs.
**Spread of dementia-specific inpatient care services for people with dementia**	In individual regions
**Expert notes regarding inpatient care**	There are only a few specialised services for people with dementia. Psychogeriatric facilities do exist in large emergency hospitals. However, the major problem is that there is a lack of staff qualified to treat people with dementia and specialised facilities such as dementia units. As people with dementia are often placed together with younger people and cared for by staff that is not trained in dementia care, they do not receive dementia-specific care in most hospitals.
**Expert conclusion regarding dementia-specific care structures**	In Romania, although there are a few public facilities (especially hospitals), most care homes are operated by private organisations. Due to the high costs of these services, which are in principle available to all, many older people with dementia do not have access to them. Further, only a few care homes provide specific services for people with dementia, and the organisations that do so rarely take their needs into account. Information on dementia is available online, but access to it is very unevenly distributed. There are some highly qualified professionals and experts who deal with the topic of dementia in detail, have a great deal of knowledge, and share important information, for example, in lectures. However, this information does not reach the majority of the population and, in particular, not the large number of people with dementia and their relatives. Many older people (especially in rural areas) do not have any internet skills, have no technical access to the internet, and lack the financial resources to participate in education. The political will to change this does not exist. Overall, a national strategy for dementia care is lacking.
**Existing models of good practise**	1. The annual events organised by the ‘Societatea Română Alzheimer’ with people with dementia and their family members, 2. some further projects initiated in different regions and by local Alzheimer Societies with self-help groups, educational programmes, theatre and storytelling groups, and other services for people with dementia, 3. awareness raising of dementia in the general population through large public campaigns

**Table 17:** Sweden

Population (2020)	10.4 million [26]
**Area (2020)**	407,300 km² [26]
**Spread of dementia-specific outpatient care services for people with dementia**	Nationwide
**Spread of dementia-specific inpatient care services for people with dementia**	Nationwide
**Expert notes regarding outpatient and inpatient care**	There are several nationally available opportunities to access dementia-specific care and support, both in the state, community, and private sectors.
**Expert conclusion regarding dementia-specific care structures**	Care services are suitable for adequate care for people with dementia and informational services for people with dementia and their family members are available nationwide. However, people with dementia and their relatives rarely participate in the development of care services. Although there are associations of relatives that try to influence dementia care, there are large regional differences.
**Existing models of good practise**	1. Different websites with information about dementia, care, and research, 2. specialised services such as dementia nurses and family support, 3. a register for people with behavioural and psychological symptoms of dementia (BPSD) that helps staff and relatives to identify different needs or symptoms in patients

**Table 18:** The UK

Population (2020)	67.1 million [27]
**Area (2021)**	243,600 km² [21]
**Spread of dementia-specific outpatient care services for people with dementia**	Nationwide
**Spread of dementia-specific inpatient care services for people with dementia**	Nationwide
**Expert notes regarding outpatient and inpatient care**	Both outpatient and inpatient care services for people with dementia are available nationwide, although most care is provided by families.
**Expert notes regarding dementia-specific information services**	Information is available throughout the country for people with dementia and their family members. However, obtaining the right information at the right time can be a challenge.
**Existing models of good practise**	An initiative that tries to bring specialists together in one place and to centralise the treatment of people with dementia. In this way, people with dementia in GP practises have access to a neurologist, for example.

### A comparative analysis of european countries

In this section, the results of the country-specific interviews with experts from 17 European countries are brought together, and an attempt is made to draw a picture regarding the existing care structures and gaps at the European level.

#### Existing structures in dementia care

**Fig. 1:**
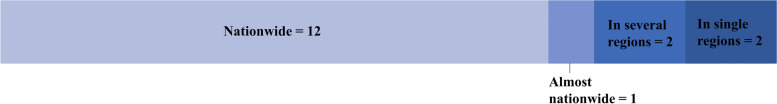
Availability of outpatient healthcare services for people with dementia

**Fig. 2:**
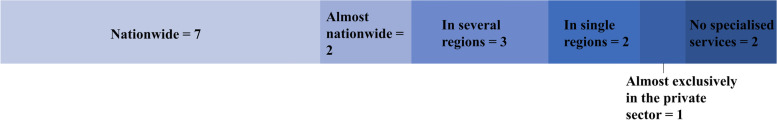
Availability of inpatient healthcare services for people with dementia

As shown in Fig. 1, the expert interviews indicated that in 13 of the 17 European countries considered, outpatient healthcare services for people with dementia are available nationwide or almost nationwide. However, regional care inequalities are also evident in a number of these countries. In Austria and Germany, the provision in some areas of outpatient care in rural areas is significantly lower and more inadequate than in urban areas, and in Italy, there is a north-south difference. In contrast, the experts from Belgium, Denmark, Finland, Liechtenstein, Luxembourg, the Netherlands, Norway, Sweden, and the UK reported that there are no significant regional inequalities in outpatient care. Whereas the countries mentioned above provide dementia-specific services nationwide, Bulgaria (almost nationwide) seems to provide care for people with dementia as a part of general health services. While specific outpatient healthcare services for people with dementia are available in several regions in Ireland and Romania, this form of care is limited to individual regions in Greece and Portugal. Inpatient healthcare services for people with dementia are only available nationwide or almost nationwide in just over half of the countries (see Fig. 2). Moreover, differences and inequalities in care are also shown in some of these countries. In Bulgaria, similar to outpatient care, no dementia-specific services are provided, in Italy, there are north-south differences, and in Finland and the Netherlands, the distance to hospitals or inpatient care facilities may be greater in some (rural) regions than in others. The experts from Liechtenstein, Luxembourg, Norway, Sweden, and the UK, on the other hand, did not report such problems. In Belgium, Germany, and Austria, inpatient healthcare services exist in several regions and in Portugal and Romania in individual regions. In Greece, there are some inpatient services in the private sector but hardly any in the public sector. No specialised inpatient services for people with dementia are available in Denmark, where a different approach is taken, as well as in Ireland.

**Fig. 3:**
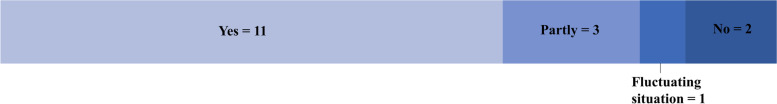
Suitability of existing services for adequate care of people with dementia

According to country-specific experts, in 11 of the 17 countries, existing care services are suitable for adequate care of people with dementia (Fig. 3), although in four countries, this is not the case (Finland, Norway, Sweden) or not to the same extent (the Netherlands) for all population groups. Furthermore, the experts from Bulgaria, point out that the existing services are quantitatively insufficient. While the expert from England could not give a clear answer for the UK due to the constantly changing situation, the experts from Belgium and Portugal indicated that this is partly the case in their countries. In Belgium, it depends on the individual organisations whether a person with dementia is offered dementia-sensitive care. In Romania, dementia-friendly services are only available in a few regions, and in Germany and Ireland, existing care services overall are not suitable for adequate care of people with dementia. The expert from Germany cited the lack of sensitivity of care providers to the specific needs of people with dementia as the main cause. In Ireland, besides the fact that existing care services are often not dementia-friendly, the problem already lies in a lack of specific services.

**Fig. 4:**
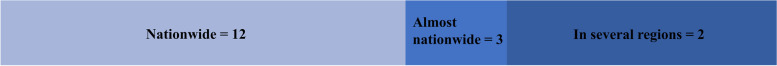
Availability of informational services for people with dementia

Informational services for people with dementia and their family members are available nationwide or almost nationwide in 15 countries, as shown in Fig. 4. However, in some of these countries, there are considerable disparities between different population groups and regions. In Germany, for example, there is a lack of specific information for people with other diverse characteristics, and in Italy, there are significantly fewer dementia-specific information services in the south of the country than in the north. The experts from the Netherlands and Romania pointed out that information is available online and that access to this information is unevenly distributed among the population (very poor access is particularly prevalent among the older rural population in Romania, who often do not have an Internet connection and/or the necessary digital skills). In Greece, on the other hand, dementia-specific information is provided nationwide through different communication channels and within different campaigns. While the experts from Luxembourg and Norway explicitly referred to the efforts of the respective national governments in terms of dementia-specific awareness and information, the Romanian expert criticized the lack of political will in Romania to do more in this area. In Belgium and Portugal, informational services for people with dementia and their family members are only provided in some regions.

**Fig. 5:**
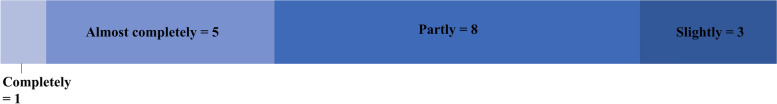
Inclusion of people with dementia into healthcare

**Fig. 6:**
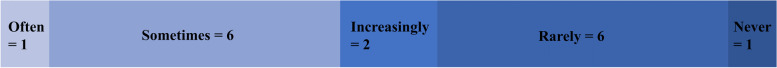
Participation of people with dementia in the development of care services

Figures 5 and 6 illustrate that the situation is different for the topics of participation and inclusion. According to the experts, people with dementia are fully (Bulgaria) or almost fully (Denmark, Finland, Italy, Liechtenstein, Luxembourg) included in healthcare in six countries. While this is still partially the case in eight countries, inclusion hardly happens in Belgium, Germany, and Norway. The only country where people with dementia or their family members are often involved in the development of care services is Finland. Such a participatory approach is sometimes adopted in Austria, Bulgaria, Ireland, Luxembourg, and the Netherlands and increasingly in Denmark and Norway. Based on expert interviews, this is rarely the case in Germany, Greece, Italy, Liechtenstein, Sweden, and the UK, and not at all in Portugal. The experts from Greece and Sweden indicate that there are some efforts in the area of involvement of people with dementia and their family members (Greece: the NDP calls for participation of people with dementia in everything related to dementia, Sweden: some associations of relatives try to influence dementia care), but this has not resulted in more participation in the development of dementia-specific care services.

## Models of good practise and existing care gaps

**Fig. 7:**
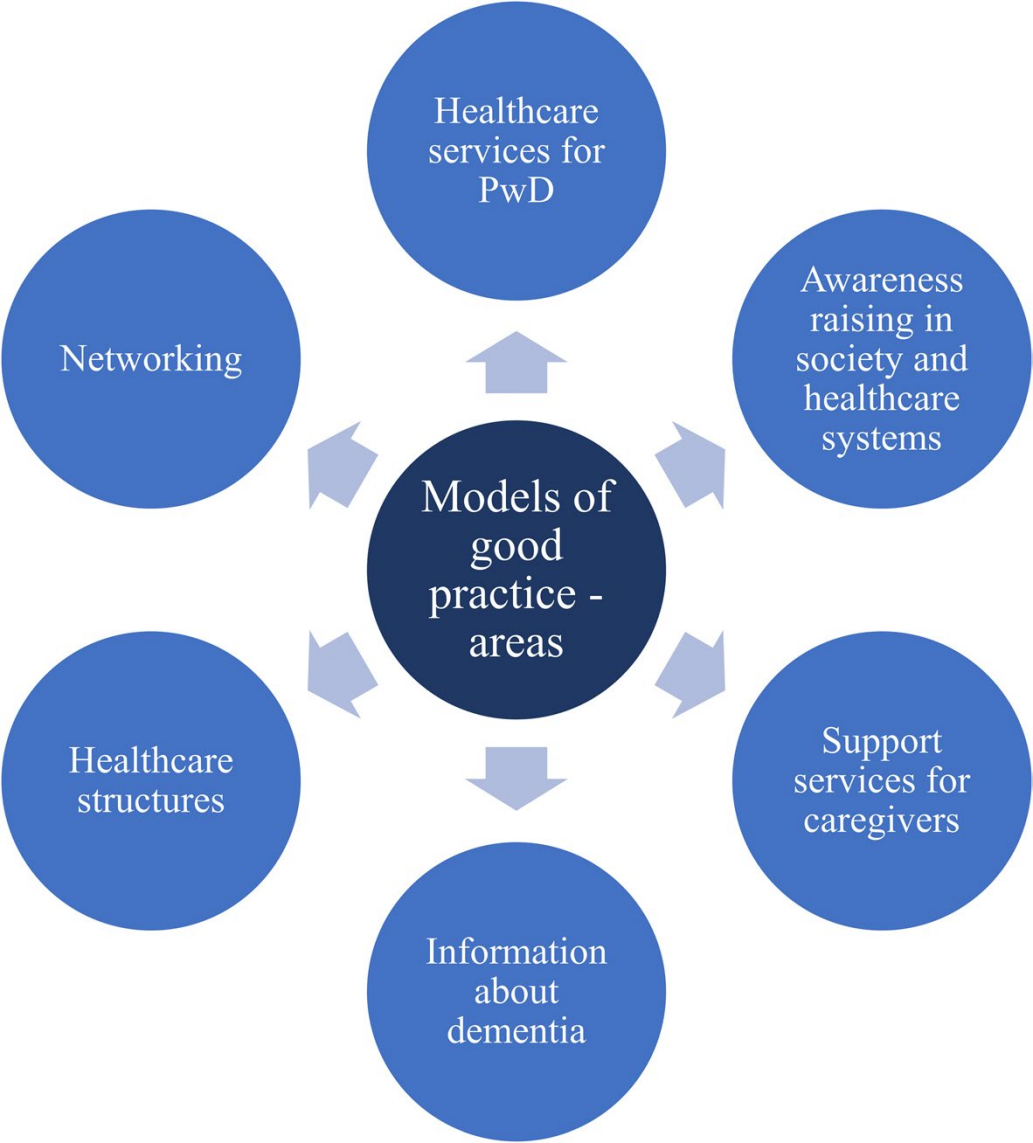
Models of good practise

Although there are considerable country-specific differences with regard to the availability and spread of dementia-specific care and informational services, as well as the inclusion and participation of people with dementia and their family members, all experts cited models of good practise in one or more of these areas (see Fig. 7). Such models were most frequently identified in the field of care services for people with dementia (eight times). For example, experts from Italy and the Netherlands referred to a high number of memory clinics, while other interviewees highlighted outreach services such as home care nurses (Austria), care, and support services (Liechtenstein), as well as dementia nurses and family support (Sweden). In Bulgaria, the concept of Alzheimer cafés is partly implemented. Another focus is on raising awareness in society and the care system (seven models of good practise). In Norway and Romania, for example, there are broad public campaigns on the topic of dementia. The Netherlands is focusing on the concept of a dementia-friendly community, and in Belgium, Denmark, and Germany, there are projects on dementia-friendly hospitals/nursing homes. Five models of good practise were cited by experts related to support services for family members. The focus here is on education, counselling, and training for family members of people with dementia (Bulgaria, Ireland, Liechtenstein, Norway). Experts from each of the three countries underscored models of good practise in the areas of dementia-specific information (Austria, Luxembourg, Sweden) and care structures (Denmark, Norway, Sweden). Especially in Denmark and Norway, there seem to be great efforts at the government level to establish nationwide standards in dementia care. Experts from Germany (where there is high activity among Alzheimer societies) and the UK (where there is an initiative to bring together GPs and specialists for dementia care) also cited models of good practise that can be assigned to the area of networking among stakeholders regarding dementia.

**Fig. 8:**
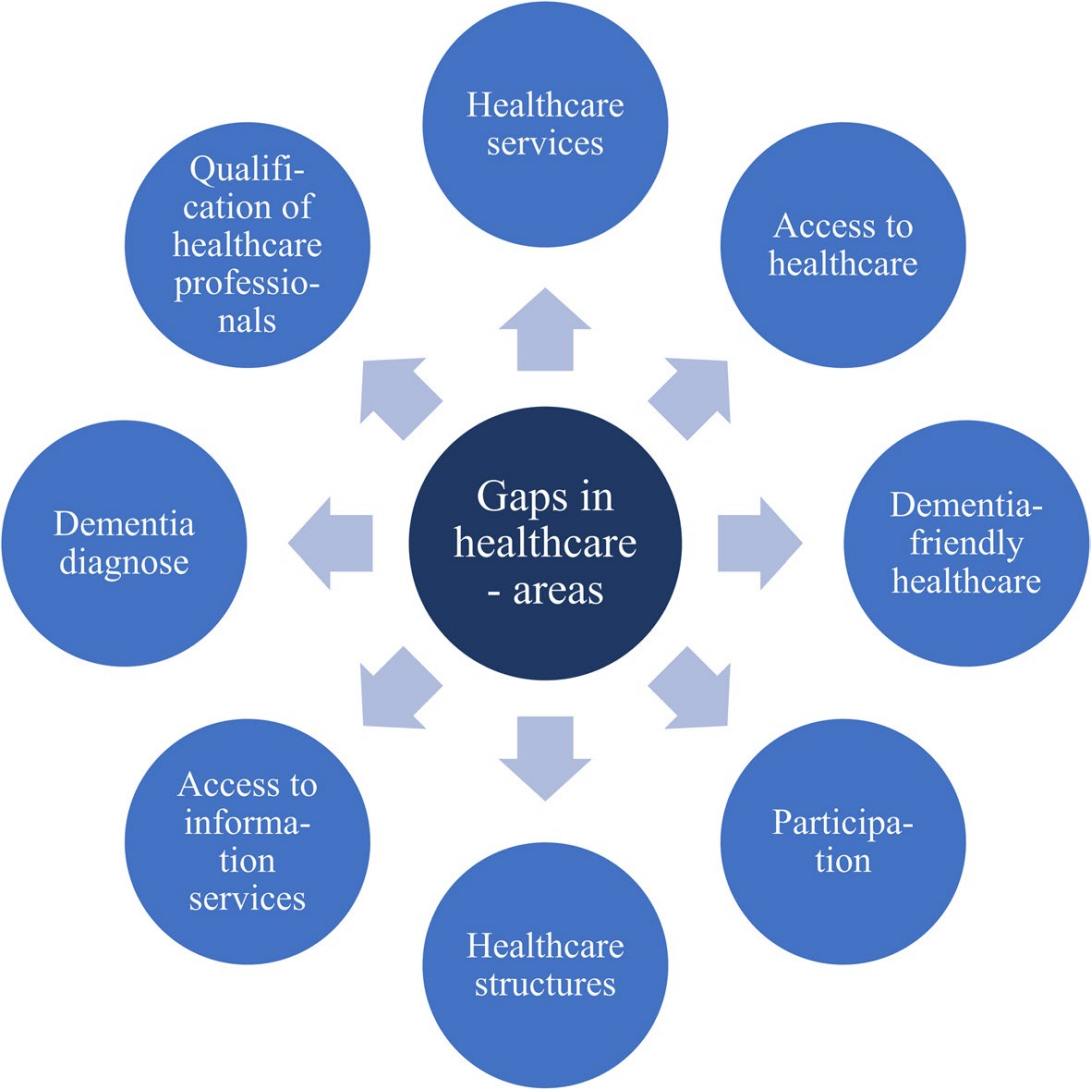
Gaps in the healthcare of people with dementia

Simultaneously, all experts pointed to structural problems and gaps regarding the healthcare of people with dementia and their relatives (the main areas are shown in Fig. 8). However, these problems and gaps seem significantly larger in some countries (Belgium, Greece, Ireland, Portugal, Romania) than in others (Finland, Liechtenstein, Luxembourg, the Netherlands, Norway, Sweden, the UK), indicating the existence of major care inequalities within Europe and the EU.

Most frequently, the interviewed experts referred to gaps in service provision for people with dementia (n = 24, see Table 19). The interviewees from Austria, Bulgaria, Germany, Portugal, and Romania emphasised that not enough dementia-specific services are available for people with dementia in both the outpatient and inpatient sectors. In Austria, Belgium, Bulgaria, Norway, and Portugal, there are significant regional differences (including a strong urban-rural contrast) in the provision of inpatient care, and in Bulgaria, Norway, Portugal, and Romania, there are large disparities in the outpatient context. Further, the experts from Germany and Romania stated that care for people with dementia in hospitals is often insufficient. In Belgium and Bulgaria, a key problem seems to be the low availability of daycare facilities. Seven of the problems described can be assigned to the area of care access. Experts from Bulgaria, Finland, and the Netherlands reported that in some parts of their country (especially in rural areas), access to care is limited. For Finland, Greece, Norway, and Portugal, the experts identified fundamental barriers to accessing healthcare for people with dementia and their families, such as bureaucratic obstacles (Norway), the high cost of services (Greece), and a lack of support (Finland). Seven of the structural gaps cited by the experts were related to the adequacy and dementia-friendliness of care services. In Belgium, Germany, and Ireland, there seems to be a lack of sensitivity of services tailored to the specific needs of people with dementia. Another area where a great need for action exists is the participation of people with dementia and their relatives in care. According to the experts, such patient and family involvement either does not happen at all or hardly takes place in Germany, Greece, Italy, Liechtenstein, Portugal, Sweden, and the UK. Six points of criticism relate to the organisation and structuring of the care system. The experts from Belgium and Portugal stated that there is a lack of financial and government support for the establishment of comprehensive care standards. The Dutch experts criticised the one-sided design of dementia care, which is determined by experts, and the Norwegian interviewee considered the implementation of existing dementia-specific knowledge in practise to require improvement. While the availability of dementia-specific information is nearly nationwide in almost all countries included, the lack of accessibility of these services is a problem in several states. For Belgium, Germany, Ireland, and Romania, the experts identified structural deficiencies in the organisation and accessibility of informational services. Two experts each pointed to difficulties in dementia diagnosis (Ireland, the Netherlands) and a major lack of dementia-specific trained staff in hospitals (Romania) or care (Belgium).

**Table 19:** Identified gaps in service provision for people with dementia

Described problem	Number (names) of countries
Not enough specific offers in outpatient care	5 (Austria, Bulgaria, Germany, Portugal, Romania)
Not enough specific offers in inpatient care	5 (Austria, Bulgaria, Germany, Portugal, Romania)
Regional differences in the provision of outpatient care	4 (Bulgaria, Norway (urban-rural divide), Portugal, Romania)
Regional differences in the provision of inpatient care	4 (Belgium, Bulgaria, Norway (urban-rural divide), Portugal (nursing homes only in larger cities), Romania)
Care for people with dementia in hospitals is often inadequate	2 (Germany, Romania)
Low availability of daycare facilities	2 (Belgium, Bulgaria)
No specific offers for people with dementia	1 (Ireland)
GP, geriatric, and rehabilitative care is not available nationwide	1 (Germany)

## Summary

Overall, efforts for dementia-friendly care and models of good care practise exist in all 17 countries. However, there are great differences between individual European countries regarding the spread of dementia-specific services and the development of structures for the care of people with dementia and their relatives. In some countries, comprehensive care structures already exist, while in other ones, care services are only available sporadically, efforts are made exclusively at the level of individual service providers or professionals, and there is a lack of political will to establish care standards. Simultaneously, in all countries, there are areas where there are major gaps in care and an urgent need for action. While salient differences are evident in the extent of the gaps, there is also some overlap in the thematic priorities between different European nations. Several experts called for restructuring measures in their country, such as a consistent national strategy for dementia care (Romania), a different structuring of the care system (Germany), or an expansion of existing structures (Austria). This could be used as a starting point for transnational networking of dementia care and the development of European strategies to establish minimum standards in the care of people with dementia and their relatives.

## Discussion

The aim of this analysis was to provide insights into existing care structures, gaps, and models of good practise in dementia-specific care based on interviews with dementia care experts from 17 European countries.

Despite some positive results in this analysis (such as the organisation of dementia care in Denmark), there are nevertheless deficits that can overlap across states. One area that was often criticised by the experts was the provision of healthcare services for people with dementia. This is reflected in the literature [28–30]. Therefore, dementia-specific care needs to be adapted accordingly. In its Alzheimer’s Innovation Readiness Index of 2021, ‘Alzheimer’s Disease International’ emphasised that care for people with dementia ‘will require a more comprehensive infrastructure for detection, monitoring, diagnosis, treatment, and care, along with more advanced legislation and policies for effectively protecting informal carers and the rights of persons living with dementia, and facilitating greater access to services and treatments’, and that both informal and professional caregivers need more dementia-specific training. However, the reality shows that corresponding initiatives can be underfunded and in part not optimally executed [28]. Broda et al. (2017) indicated that there are different components for optimised dementia care, e.g. multidisciplinary, person-centred care services, the need to ease the dementia care pathway, and the networking of formal and informal care [31]. Alzheimer Europe highlighted the importance of dementia friendliness and the need to build a model for dementia-friendly communities that could be implemented on a European basis. This model should integrate existing efforts at the national level but leave room for individual adaptation to local circumstances [32].

Another area where significant deficits are evident is the participation of people with dementia and family carers in the development of healthcare services and information. Although greater awareness and understanding of the importance of the participation of people with dementia in healthcare services, research, and support have emerged in recent decades [33], the present analysis revealed a need for improvement. It is of immense relevance to involve people with dementia in the creation of healthcare services (as well as research and policy) for the following reasons: (1) people with dementia are experts in dementia and involving them leads to better outcomes; (2) involving them has advantages for people with dementia themselves, but also for (healthcare) professionals; and (3) people with dementia have the right to be involved. Thus, the participation of people with dementia and their family carers in the design and evaluation of healthcare services leads to services that are better tailored to their needs [33]. There are different ways of involving people with dementia. A scoping review from 2019 on design research reports on methods, recruitment, and tools among others when trying to involve people with dementia. It is suggested that one could conduct workshops together with people with dementia in which the needs of people with dementia could be identified, services or interventions could be created, or feedback could be obtained [34]. Kort et al. (2019) report on methods of involving people with dementia from five different projects. These include: observation, consultation, storytelling, focus group sessions, thinking-aloud sessions, and photo production and interviews [35]. A good example of people with dementia participating in designing information material is the ‘Living Well Handbook’. In this handbook, people with dementia and their carers note important information, such as emergency details, relevant facts about the person with dementia, and care planning. It further contains lists organisations that hand out information on dementia, provide support, plan future steps, and provide the help a person with dementia might need. This handbook was developed together with people with dementia and their carers [36].

When doing research on dementia and including people with dementia in research and in the design of information and healthcare services it is important to not only focus on people with dementia in general. It is relevant to pay special attention to people with dementia that are younger or come from the LGBTQIA + community or have a migration background or belong to an ethnic minority group. These are populations that not only face the dementia-specific challenges but also encounter other differences, such as aggravated access to healthcare for people from ethnic minorities, heteronormative healthcare services that are not suitable for people from the LGBTQIA + community, lack of healthcare services for younger people with dementia, and therefore have additional needs [13, 37, 38].

A further important aspect is the role of governments in the care of people with dementia and family carers. First, it is vital that they recognise dementia as a public health priority, which is not yet the case in all European countries [39]. In addition, they have a special responsibility to improve access to and quality of care, to help (in)formal caregivers, and to ease care pathways for those affected [28].

These are only a few relevant aspects regarding the care of people with dementia. Coordinating, repositioning, and optimising care for People with dementia and family carers requires a combination of actions among key stakeholders such as care providers, care planners, patient organisations, research, and governments.

## Limitations

A limitation regarding the generalisability of this study’s findings is that the group of interview participants must be described as selective and not representative of experts in dementia care in European countries. Despite an extensive search, it was not possible to include an expert for every EU, EFTA, and UK country. While several experts were identified and interviewed in a few states, only one expert could be recruited in most of the countries studied. Moreover, according to self-assessment, not all interview participants were primarily experts in the field of dementia. Some participants focused on general healthcare or the topic of migration (e.g. Greece), which is partly because experts in the area of dementia and migration were originally searched for. Furthermore, the experts have different professional backgrounds, which poses a limitation for the comparability of the results. Hence, the findings do not provide a complete picture of the care situation in individual European countries, although that was not the aim. Rather, this paper is intended to draw attention to specific country-specific and cross-country care areas where there is a particular need for action. This study can and should be used as a starting point for further, more in-depth analyses with experts from different areas of formal and informal care in as many European states as possible. For a valid description of the situation regarding the formal care of people with dementia and their relatives, as well as existing specific care services for this population in individual European countries, more research is necessary using different methods, such as country-specific literature analyses and large-scale surveys among care experts, planners, and providers in the respective countries.

The interview guide designed for the project ‘EU-Atlas - Prevalence of dementia in people with a migration background’ contains specific questions on the care of people with dementia with a migratory background [12]. According to the authors, the general questions on dementia-specific care structures are sufficient to give initial insights into basic inequalities and gaps in the care systems and to identify individual models of good care practise, but an objective and comprehensive representation of care services cannot be provided. The interview guide and the results were shaped by the experts’ points of view. To reduce the influence of the individual’s personal opinion and to improve the quality of the answers, the guide was sent to the experts some time before the interviews were held. Another limitation concerns the predefined answer categories, which improved the comparability of the data but distorted the experts’ documented views on the care situation in their respective countries, and led to an underestimation of complexities in individual nations. To counteract this, open questions were asked after almost every closed question. Despite these limitations, the study provides useful information for care planners at the national and European levels regarding current problems in formal dementia care, existing gaps in European health systems, important dementia-specific fields of action, and models already established in individual countries to address the problems described. This study is a valuable addition to the current literature on existing structures for the care of people with dementia in Europe, as its mixed-methods approach and the consultation of experts in care practise from individual countries provide more in-depth, country-specific information and first-hand conclusions, and can thus be used as a reference point for comparisons between care planning written down in documents and the actual circumstances in care practise.

## Conclusion

The considerable inequalities in dementia care within and between many European countries, as well as the structural deficiencies in key areas of care that exist to varying degrees in all countries and partly show large overlaps (according to interviews with country-specific experts from 17 EU, EFTA, and UK countries), illustrate the need for major restructuring measures and paradigm shifts. Changes like these are, of course, dependent on the individual healthcare systems in each country. What can be done in one country might not be feasible and manageable in another. However, to initiate such extensive systemic transformations and to establish quality-related minimum standards in the care of people with dementia—which is often marginalised and has specific needs—supraregional and (ideally) transnational strategies are required. Since the EU was originally a community of values and its institutions are committed to the goal of achieving equal living conditions for all population groups living in its member states, it should be the EU’s task to develop such concepts at the European level together with cooperation partners, such as the parliaments and ministries of their member states, care planners and experts, research institutions, care providers, and organisations of people with dementia and their relatives. Within the framework of EU programmes such as European care guidelines or dementia plans, which must receive binding budgeting in addition to concrete time schedules, special attention should be given to the European networking of key stakeholders in dementia care; the sensitisation of European societies and care systems to the topic of dementia; the development of Europe-wide care structures for people with dementia and their relatives in both outpatient and inpatient contexts; the validation of the dementia-friendliness of these services with people with dementia and their relatives; the elimination of barriers for the access of people with dementia and their relatives to information, support, and care services; the dementia-specific education and training of health and care professionals; and the participation of people with dementia and their relatives in all areas of dementia-specific care and research. In particular, the inclusion of people with dementia and their relatives in European societies and care systems should be given greater priority in future dementia care strategies. In addition to a fundamentally open attitude toward the needs of other people, adopting a supraregional and transnational perspective can be helpful in this regard. Some existing models of good practise, such as the Norwegian dementia action plan or the concepts of dementia-friendly communities, hospitals, or nursing homes, which have been partially implemented in Belgium, Denmark, Germany, and the Netherlands, can be used as a starting point for broadening the perspective and for international cooperation.

## Supplementary Information

**Additional file 1.**

## Data Availability

The data are not publicly available because the answers to the questions asked are personal assessments of experts working in the care or research system of the respective countries, which have been anonymised in this article. For data, please contact Jessica Monsees (jessica.monsees@dzne.de).

## Abbreviations

EFTA: European Free Trade Association
EU: European Union
NDPs: National dementia plans
UK: United Kingdom

## Footnotes

1 European Union.

2 European Free Trade Association.

3 United Kingdom.

## References

[1] ICD-10-GM, Version. 2021. 2021. https://www.dimdi.de/static/de/klassifikationen/icd/icd-10-gm/kode-suche/htmlgm2021/block-f00-f09.htm. Accessed 14 Dec 2021.

[2] Leung DKY, Chan WC, Spector A, Wong GHY (2021). Prevalence of depression, anxiety, and apathy symptoms across dementia stages: a systematic review and meta-analysis. Int J Geriatr Psychiatry.

[3] Black BS, Johnston D, Leoutsakos J, Reuland M, Kelly J, Amjad H, Davis K, Willink A, Sloan D, Lyketsos C, Samus QM (2019). Unmet needs in community-living persons with dementia are common, often non-medical and related to patient and caregiver characteristics. Int Psychogeriatr.

[4] Eichler T, Thyrian JR, Hertel J, Richter S, Wucherer D, Michalowsky B, Teipel S, Kilimann I, Dreier A, Hoffmann W (2016). Unmet Needs of Community-Dwelling Primary Care Patients with Dementia in Germany: Prevalence and Correlates. J Alzheimers Dis.

[5] Chiao C-Y, Wu H-S, Hsiao C-Y (2015). Caregiver burden for informal caregivers of patients with dementia: a systematic review. Int Nurs Rev.

[6] Zwingmann I, Hoffmann W, Michalowsky B, Wucherer D, Eichler T, Teipel S, Dreier-Wolfgramm A, Kilimann I, Thyrian JR (2018). Offene Versorgungsbedarfe pflegender Angehöriger von Menschen mit Demenz. Nervenarzt.

[7] Zwingmann I, Michalowsky B, Esser A, Kaczynski A, Monsees J, Keller A, Hertel J, Wucherer D, Thyrian JR, Eichler T (2019). Identifying Unmet Needs of Family Dementia Caregivers: Results of the Baseline Assessment of a Cluster-Randomized Controlled Intervention Trial. J Alzheimers Dis.

[8] Alzheimer Europe (2019). Dementia in Europe Yearbook 2019. Estimating the prevalence of dementia in Europe.

[9] Cimler R, Maresova P, Kuhnova J, Kuca K (2019). Predictions of Alheimer’s disease treatment and care costs in European countries. PLoS One.

[10] Alzheimer Europe (2018). Dementia in Europe Yearbook 2018: comparison of national dementia strategies in Europe. In.

[11] Europäische Kommission (2021). Gesundheitspolitische Strategie - Überblick.

[12] Monsees J, Schmachtenberg T, Leitz M, Cardona MI, Stentzel U, van den Berg N, Hoffmann W, Thyrian JR (2021). EU-Atlas. Dementia & Migration: estimated number, care situation, and policies regarding people with a migration background with dementia in the EU, EFTA, and UK.

[13] Alzheimer Europe (2018). The development of intercultural care and support for people with dementia from minority ethnic groups.

[14] Kuckartz U (2010). Einführung in die computergestützte Analyse qualitativer Daten.

[15] Mayring P (2014). Qualitative Content Analysis: Theoretical Foundation, Basic Procedures and Software Solution.

[16] Statistisches Bundesamt (2021). Eurostat-Daten: Österreich im Vergleich.

[17] Statista (2021). Fläche von Österreich nach Bundesländern.

[18] Statistisches Bundesamt (2021). Eurostat-Daten: Belgien im Vergleich.

[19] Wirtschaftskammer Österreich (2021). Fläche und Bevölkerung.

[20] Eurostat (2021). Population on 1 January by age and sex.

[21] Statista (2021). Europäische Union: Flächen der Mitgliedsstaaten im Jahr 2021.

[22] Statistisches Bundesamt (2021). Eurostat-Daten: Finnland im Vergleich.

[23] Statistisches Bundesamt (2021). Bevölkerungsstand: Amtliche Einwohnerzahl Deutschlands 2021.

[24] Amt für Statistik Fürstentum Liechtenstein (2021). Liechtenstein in Zahlen 2021.

[25] Auswärtiges A (2021). llgemeine Landesinformationen: Norwegen.

[26] Statistisches Bundesamt (2021). Schweden: Statistisches Länderprofil.

[27] Office for National Statistics (2021). Population estimates for the UK, England and Wales, Scotland and Northern Ireland: mid-2020.

[28] Alzheimer’s Disease International (2021). 2021 Alzheimer’s Innovation Readiness Index.

[29] Alzheimer Europe (2021). European Dementia Monitor 2020. Comparing and benchmarking national dementia strategies and policies.

[30] Martin A, O’Connor S, Jackson C (2020). A scoping review of gaps and priorities in dementia care in Europe. Dementia (London).

[31] Broda A, Bieber A, Meyer G, Hopper L, Joyce R, Irving K, Zanetti O, Portolani E, Kerpershoek L, Verhey F (2017). Perspectives of policy and political decision makers on access to formal dementia care: expert interviews in eight European countries. BMC Health Serv Res.

[32] Alzheimer Europe (2015). Dementia in Europe Yearbook 2015. “Is Europe becoming more dementia friendly?“.

[33] Skiadzien E (2021). Valuing the advocacy of people with dementia: moving dementia out of the shadows.

[34] Wang G, Marradi C, Albayrak A, van der Cammen TJM (2019). Co-designing with people with dementia: A scoping review of involving people with dementia in design research. Maturitas.

[35] Kort HSM, Steunenberg B, van Hoof J (2019). Methods for Involving People Living with Dementia and Their Informal Carers as Co-Developers of Technological Solutions. Dement Geriatr Cogn Disord.

[36] Gloucestershire NHS (2013). Living Well Handbook.

[37] Rimkeit S, McIntosh J (2017). Experiencing place: younger people with dementia facing aged care. Australasian Psychiatry.

[38] Harper P (2019). How healthcare professionals can support older LGBTQ + people living with dementia. Nurs Older People..

[39] Alzheimer Europe (2017). Dementia in Europe Yearbook 2017. Standards for residential care facilities in Europe.

