# Supplementary material for: Structures for the care of people with dementia: a European comparison
Source: BMC Health Serv Res. 2022 Nov 18;22:1372. doi: 10.1186/s12913-022-08715-7 (PMC9673874; doi:10.1186/s12913-022-08715-7)
Supplement: Supplementary file 1 — Additional file 1. [file 12913_2022_8715_MOESM1_ESM.docx]

**Supplementary information**

**List of questions considered in this study**

1. Please estimate to what extent are services for outpatient care of people with dementia widespread in your country?

□ Nationwide

□ Almost nationwide

□ In several regions (areas)

□ In single regions

□ Not available

- 1. Could you explain this in more detail?

1. Please estimate how widely available services for inpatient care of people with dementia are in your country?

□ Nationwide

□ Almost nationwide

□ In several regions (areas)

□ In single regions

□ Not available

- 1. Could you explain this in more detail?

1. According to your opinion, are existing services suitable for the adequate care of people with dementia?

□ Yes, for people with and without a migratory background

□ Yes, but only for people without a migratory background

□ Yes, but only for people with a migratory background

□ No, neither for people without nor for people with a migratory background

1. Could you explain this in more detail?
2. Could you please estimate the level of inclusion of people with dementia into healthcare in your country?

□ Completely

□ Almost completely

□ Partly

□ Slightly

□ Not at all

- 1. Are there any models of good practise?

1. According to your opinion, how widely available are informational services for people with dementia and their family members in your country?

□ Nationwide

□ Almost nationwide

□ In several regions (areas)

□ In single regions

□ Not existent/available

- 1. Could you explain this in more detail?

1. According to your opinion, are people with dementia and/or their family members participating in the development of healthcare services, the design of informational material, or the design of residential and care facilities?

□ Always

□ Often

□ Sometimes

□ Rarely

□ Never

- 1. Could you explain this in more detail?
